# Discovery of a drug targeting microenvironmental support for lymphoma cells by screening using patient-derived xenograft cells

**DOI:** 10.1038/srep13054

**Published:** 2015-08-17

**Authors:** Keiki Sugimoto, Fumihiko Hayakawa, Satoko Shimada, Takanobu Morishita, Kazuyuki Shimada, Tomoya Katakai, Akihiro Tomita, Hitoshi Kiyoi, Tomoki Naoe

**Affiliations:** 1 Department of Hematology and Oncology, Nagoya University Graduate School of Medicine, Nagoya, Japan; 2 Fujii Memorial Research Institute, Otsuka Pharmaceutical Co., Ltd., Otsu, Japan; 3 Department of Pathology and Clinical Laboratories, Nagoya University Hospital, Nagoya, Japan; 4 Department of Immunology, Niigata University Graduate Scholl of Medical and Dental Sciences; 5 National Hospital Organization Nagoya Medical Center, Nagoya, Japan

## Abstract

Cell lines have been used for drug discovery as useful models of cancers; however, they do not recapitulate cancers faithfully, especially in the points of rapid growth rate and microenvironment independency. Consequently, the majority of conventional anti-cancer drugs are less sensitive to slow growing cells and do not target microenvironmental support, although most primary cancer cells grow slower than cell lines and depend on microenvironmental support. Here, we developed a novel high throughput drug screening system using patient-derived xenograft (PDX) cells of lymphoma that maintained primary cancer cell phenotype more than cell lines. The library containing 2613 known pharmacologically active substance and off-patent drugs were screened by this system. We could find many compounds showing higher cytotoxicity than conventional anti-tumor drugs. Especially, pyruvinium pamoate showed the highest activity and its strong anti-tumor effect was confirmed also *in vivo*. We extensively investigated its mechanism of action and found that it inhibited glutathione supply from stromal cells to lymphoma cells, implying the importance of the stromal protection from oxidative stress for lymphoma cell survival and a new therapeutic strategy for lymphoma. Our system introduces a primary cancer cell phenotype into cell-based phenotype screening and sheds new light on anti-cancer drug development.

Most anti-cancer drug developments today are adopting either target-based screening or cell-based phenotypic screening to identify potential compounds. Target-based screening is a powerful tool if the targeted cancer relies on a specific driver mutation, such as *BCR-ABL* in chronic myeloid leukemia^1^ and *EML4-ALK* in non-small-cell lung cancer^2^; however, many cancers do not depend on a single mutation or a growth signal and target-based screening resulted in reduced success in discovering anti-cancer drugs due to drug resistance by clonal evolution and alternative growth signal activation in cancer cells^3^,^4^,^5^. On the other hand, it is important for phenotypic screening that the screening system recapitulates the disease pathology. For the development of anti-cancer drugs, it has been usual to measure the growth inhibitory effect on established cancer cell lines; however, cancer cell lines do not recapitulate cancer pathology in some aspects. Most cell lines are quite different from primary tumor cells in the points of microenvironment-independent survival and rapid growth^6^. These gaps could be the reason for the failure of a clinical trial because of an insufficient anti-tumor effect despite the high anti-tumor activity of the drug in a pre-clinical study using cell lines. Survival support from the microenvironment may confer unexpected drug resistance on cancer cells^7^. In addition, drugs picked up by cell line-based screening tend to be more sensitive to rapid-growing cells and may be less sensitive to slow-growing primary tumors, especially cancer stem cells. Most cell line-based screening cannot target such microenvironment-dependent survival support^6^,^8^. Using primary tumor cells for screening can be a solution; however, it is difficult to perform for the following reasons: 1) primary cancer cells are not suitable for analyses of the growth inhibitory effect or cytotoxicity, because they cannot survive in *ex vivo* culture, especially after thawing frozen cells; 2) it is difficult to set up a large-scale screening because fresh primary human cancer cells are difficult to obtain at the desired time; 3) due to the limitation of the obtained cell number and preservation, a large-scale screening and repeated screening to confirm reproducibility are difficult. As a solution to these problems, we developed a new drug-screening system using lymphoma cells obtained from patient-derived xenografts (PDX) that established by the transfer of primary cancer cells directly from patients into immunodeficient mice. PDX could provide primary-like lymphoma cells of the needed amount at the desired time. We developed a method for *ex vivo* culture that could maintain their phenotype and applied it to a high throughput screening system. The selected compound demonstrated high anti-tumor activity both *ex vivo* and in a mouse model and had a totally different mechanism of action from conventional anti-tumor drugs, inhibition of glutathione supply from stromal cells to lymphoma cells. Our system introduces a primary cancer cell phenotype into cell-based phenotype screening and sheds new light on anti-cancer drug development.

## Results

### Establishment lymphoma PDX

We first established PDX by transplanting primary lymphoma cells into NOD/SCID IL-2Rγc^−/−^ (NOG) mice. Lymphoma cells were collected from patients with informed consent. This study was approved by the institutional review board of Nagoya University Graduate School of Medicine. We finally established 4 PDX, 3 diffuse large B cell lymphoma (DLBCL) and one intravascular lymphoma. Patients’ characteristics are shown in Supplemental Table 1. All models were confirmed to be serially transplantable. Lymphoma cells of 8–70 × 10^6^ were obtained from a mouse 7–10 weeks after transplantation. We designated these lymphoma cells as PDX cells. Global gene expression profiles of PDX cells showed high similarity to those of original primary cells. The correlation coefficient of gene expression profiles between PDX cells and the original primary cells was 0.814–0.890. These data are summarized in Supplemental Table 2.

**Figure 1:**
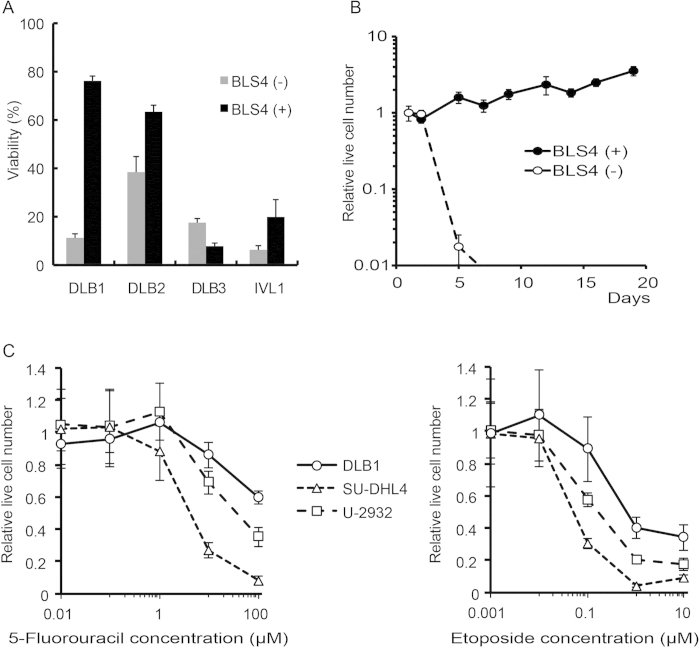
Establishment of *ex vivo* culture of PDX cells. (**A**) Co-culture with BLS4 inhibited cell death of PDX cells in some cases. Indicated PDX cells (3 × 10^5^/ well) were cultured with or without BLS4 (3 × 10^4^/ well) in a 12-well plate for 72 h. Viability was measured by flow cytometory after DAPI staining and plotted on a bar chart. (**B**) Long-term *ex vivo* culture of PDX cells. DLB1 cells were cultured with or without BLS4. In co-culture with BLS4, DLB1 cells were stripped from BLS4 by pipetting and re-seeded on newly prepared BLS4 once a week. Live cell numbers were counted by the trypan blue exclusion method and plotted on a line graph. (**C**) DLB1 cells were more resistant to anti-tumor drugs than lymphoma cell lines. DLB1 cells co-cultured with BLS4, SU-DHL4 and U-2932 treated with the indicated concentrations of 5-fluorouracil (left graph) and etoposide (right graph) for 48 h. Live lymphoma cell numbers were counted by the propidium iodide exclusion method and relative live cell numbers were plotted on a line graph.

We next set out to culture PDX cells *ex vivo*; however, no PDX cells could survive without stromal cells. We therefore searched for stromal cells that could support the survival of PDX cells. In the putative model of the microenvironment of lymphoma, lymphoma cells are nurtured by a variety of stromal cells, such as follicular helper T cells (FTH), follicular dendritic cells (FDC) and fibroblastic reticular cells (FRC). Stromal cells secrete cytokines and chemokines beneficial for growth and survival, stimulate B cell receptor by specific antigen presentation and help to avoid the anti-tumor immune response^9^,^10^. In particular, FRC has been recently revealed to play an important role in the formation of a lymphoma-permissive niche^11^. In addition, in NOG mice, T, B and NK cells are defective and the function of dendritic cells is severely impaired, suggesting that FTH and FDC do not play a role in lymphoma cell survival support in NOG mice^12^. Therefore, we focused on FRC as survival-supporting cells in the lymphoma PDX. FRC produces reticular fibers (RF) to make a mesh-like structure, the reticular network (RN), in response to contact with lymphocytes and lymphoma cells through lymphotoxin^11^,^13^. RN supports the tissue architecture of LN and can be observed by silver staining. We examined whether RN formation occurred in the lymphoma PDX. RN formation was observed with silver staining in tumors in all lymphoma PDX (Supplemental Figure 1). These results indicated that lymphoma cells stimulated FRC to form RN also in the lymphoma PDX and that co-culture with FRC was a culture system reflecting the *in vivo* microenvironment of the lymphoma PDX. Next, we investigated whether FRC could support the survival of PDX cells *ex vivo* using a mouse FRC cell line, BLS4. Strikingly, co-culture with BLS4 inhibited cell death of 3 PDX cells out of 4 (Fig. 1A). In particular, co-culture with BLS4 demonstrated the strongest survival-supportive effect on DLB1 cells and enabled long-term culture of DLB1 cells for more than 19 days, although they grew very slowly and the doubling time was 9.85 days (Fig. 1B). Furthermore, co-culture with BLS4 maintained the global gene expression profile of DLB1 cells almost completely for at least 4 days (Supplemental Figure 2).

### PDX cells were more resistant to anti-tumor drugs than lymphoma cell lines

The slow growth rate of DLB1 cells prompted us to examine whether they were less sensitive to anti-tumor drugs than cell lines. Strikingly, DLB1 cells co-cultured with BLS4 were less sensitive to 5-fluorouracil, an anti-metabolite and etoposide, a topoisomerase II inhibitor, than SU-DHL4 and U-2932, cell lines of DLBCL (Fig. 1C). GI50 of 5-fluorouracil could not be determined for DLB1 cells, while it was 4.13 μM and 38.4 μM for SU-DHL4 and U-2932, respectively. GI50 of etoposide was 610 nM, 49 nM and 170 nM for DLB1 cells, SU-DHL4 and U-2932, respectively. These results suggested that the drug sensitivity profiles of PDX cells were different from those of cell lines.

### Establishment of PDX cell-screening

**Figure 2:**
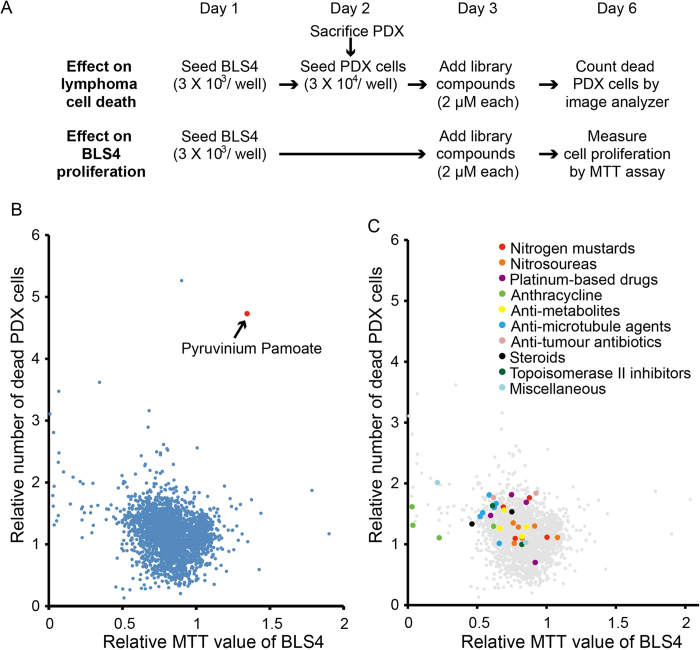
Development of PDX cell-screening. (**A**) Flowchart of PDX cell-screening. Library compounds were added to DLB1 cells co-cultured with BLS4 to see their effect on lymphoma cell death. Compounds were also added to BLS4 alone to see their effect on BLS4 proliferation. (**B**) Results of PDX cell-screening. After 72 h treatment with library compounds, the effect of compounds on lymphoma cell death were measured by counting dead PDX cells stained with DAPI by an image analyzer and the effect on BLS4 proliferation was measured by MTT assay. All compounds were plotted on a scattergram where relative numbers of dead PDX cells and relative MTT values were set on the Y-axis and X-axis, respectively. The plot position of pyruvinium pamoate is indicated by a red dot. (**C**) Plot positions of conventional anti-tumor drugs. Plot positions of conventional anti-tumor drugs in the same screening in (**B**) are indicated by a different color for each group of drugs.

It was expected that drugs effective to PDX cells had different mechanisms of action from conventional anti-tumor drugs and could be innovative anti-tumor drugs; therefore, we tried to set up a drug-screening system using PDX cells. Because DLB1 cells were the most suitable for *ex vivo* culture (Fig. 1A), we selected DLB1 cells for the screening. The flow-chart of the screening procedure is shown as Fig. 2A. PDX cells were obtained from mice on day 2 of screening and were seeded on BLS4. PDX cells were treated with the Prestwick and Lopack chemical library containing 2613 off-patent drugs and pharmacologically active compounds from day 3. After 72 h treatment, dead PDX cells were stained with DAPI and counted with an image analyzer that could distinguish PDX cells from BLS4 by cell size and selectively count dead PDX cells. We performed on MTT assay of monocultured BLS4 treated with the same chemical library to exclude compounds highly toxic to BLS4 because it could not be rejected that PDX cell death by such compounds came from the loss of survival-supporting cells independently of their cytotoxicity to PDX cells. Both assays were performed well, with coefficient of variation values of 7.87% and 7.88% and z’-factors of 0.69 and 0.69, respectively. All compounds were plotted on a scattergram where relative numbers of dead PDX cells and relative MTT values were set on the Y-axis and X-axis, respectively (Fig. 2B). Compounds plotted in the right-upper area induced strong PDX cell death and were less toxic to BLS4. The relative numbers of dead PDX cells were multiplied by the relative MTT values to calculate the drug effect index (DEI). The list of top 50 compounds with high DEI is shown in Supplemental Table 3. The compound with the highest DEI was pyruvinium pamoate (PP), an FDA-approved classical anthelminthic^14^ (Fig. 2B and Supplemental Table 3). The screening results of the 36 conventional anti-tumor drugs included in the library are shown in Supplemental Table 4 and their plot positions are indicated by different colors in Fig. 2C. Many of the conventional anti-tumor drugs did not induce PDX cell death strongly in our system and their DEI was low. The top 4 drugs were bleomycin, melphalan, carboplatin and oxaliplatin, which ranked 36th, 70th, 122nd and 200th of the 2613 compounds, respectively. These results indicated that our screening system targeted different mechanisms of anti-tumor reagents and could picked up different compounds from conventional screening. We designated this screening system as PDX cell-screening.

### PP was an effective anti-tumor drug both *ex vivo* and *in vivo*

**Figure 3:**
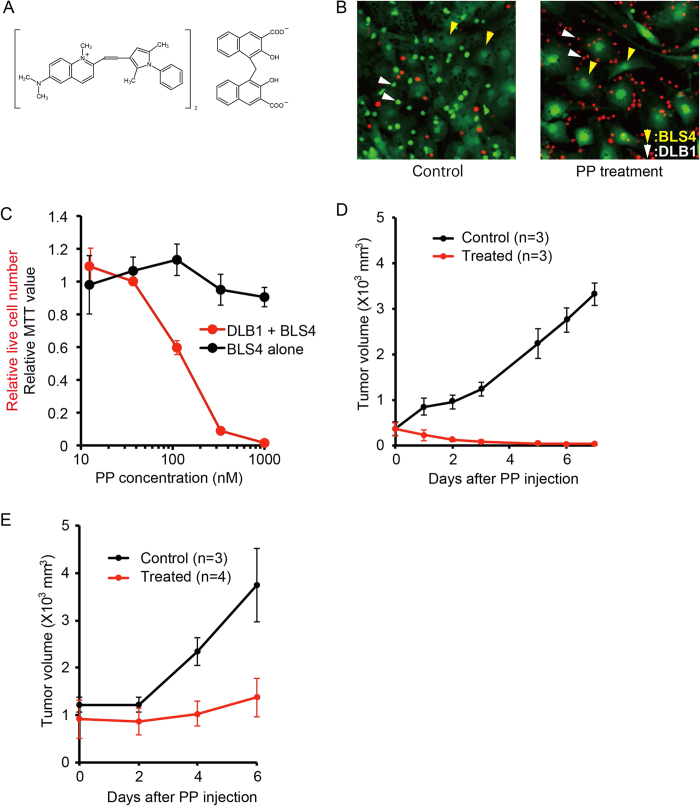
PP was an effective anti-lymphoma drug both *ex vivo* and *in vivo*. (**A**) Structure of PP. (**B**) Selective lymphoma cell death by PP. DLB1 cells co-cultured with BLS4 were treated with 1 μM PP for 48 h. Live cells and dead cells were stained with Calsein-AM (green) and PI (red), respectively. White and yellow arrowheads indicate lymphoma cells (live and dead cells) and BLS4 (live cells only), respectively. (**C**) Selective lymphoma cell death by PP. DLB1 cells co-cultured with BLS4 treated with the indicated concentrations of PP and stained as in (**B**). Live lymphoma cell numbers were counted by an image analyzer and relative live cell numbers were plotted on a line chart (red). Monocultured BLS4 were also treated with PP in the same way as co-cultured BLS4. Their proliferation was measured with MTT assay and relative MTT values were plotted on the line chart (black). (**D**) Disappearance of subcutaneous PDX cell tumor by single injection of PP in mice. DLB1 cells (5 × 10^6^) and BLS4 (2 × 10^5^) were subcutaneously inoculated into NOG mice. After subcutaneous tumors reached 300 mm^3^, mice were treated by a single intratumoral injection of 20 mg/kg PP (treated: n = 3) or 5% acasia (control: n = 3). Tumor volumes were measured and plotted on a line chart. Subcutaneous tumors had almost disappeared in the treated group 7 days after the single injection of PP. (**E**) PP induced strong tumor growth suppression in another lymphoma PDX. DLB2 cells (5 × 10^6^) and BLS4 (2 × 10^5^) were subcutaneously inoculated into NOG mice. PP administration and the measurement of the tumor volume were performed as in (**D**).

The structure of PP is shown in Fig. 3A. PP was originally approved as an anthelminthic. Recently, it attracted particular attention as an anti-tumor drug since it was revealed to have cytotoxicity against various cancer cell lines. We first confirmed the lymphoma cell-specific cytotoxicity of PP. In the co-culture of DLB1 cells with BLS4, 1 μM PP induced strong cell death, specifically of lymphoma cells (Fig. 3B). GI_50_ at 48 h of PP for DLB1 cells co-cultured with BLS4 was 0.137 μM, while even 1 μM PP did not significantly affect the viability of BLS4 and GI_50_ was not determined (Fig. 3C). Next, we examined the effect of PP on the DLB1 xenograft. PP is an anthelminthic for intestinal parasites and administered orally in humans. But its bioavailability is very poor and its systemic absorption hardly occurs in human^15^. In addition, after the intraperitoneal administration of PP at the maximum tolerated dose (5 mg/kg), the maximum serum level of PP was 99.3 nM at 15 minutes after the injection^16^, indicating that the blood concentration of PP could not reach to the level expected to be effective for lymphoma by intraperitoneal administration; therefore, we administered PP locally. PP 20 mg/kg was administered directly to the subcutaneous tumors of DLB1 cells and BLS4. A single administration was enough for tumor disappearance (Fig. 3D), demonstrating the high anti-tumor activity of PP *in vivo*. Furthermore, PP also induced strong growth suppression of the subcutaneous tumor of DLB2 cells, another lymphoma PDX cells, indicating the possible wide range effectiveness of PP for lymphoma cells (Fig. 3E).

### PP showed a strong anti-tumor effect by inhibiting glutathione supply from FRC to lymphoma cells

**Figure 4:**
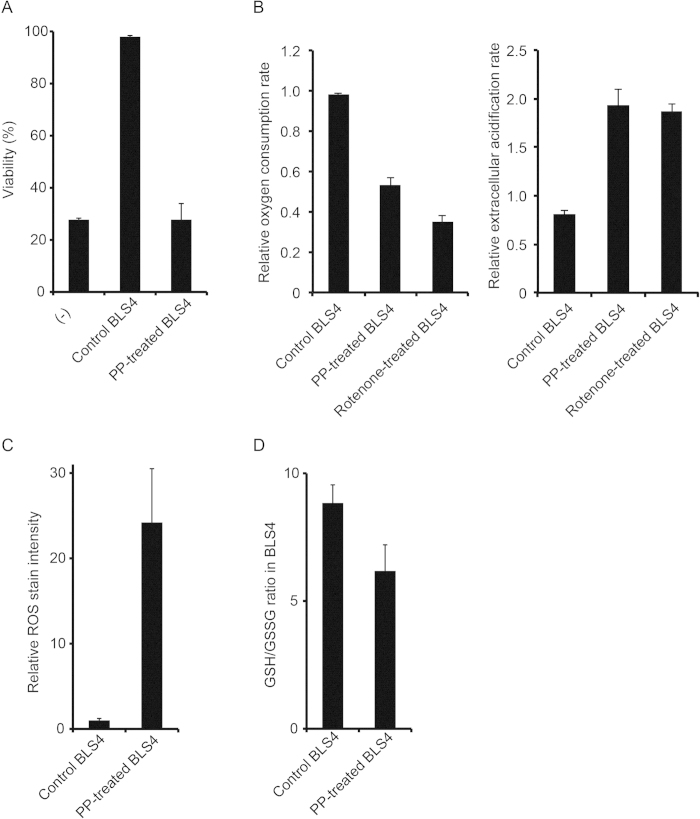
PP induced ROS production through inhibition of mitochondrial respiration in BLS4 and impaired its ability to support lymphoma cell survival. (**A**) PP-treated BLS4 lost the ability to support PDX cell survival. BLS4 (3 × 10^3^/ well) in a 96-well plate was treated with or without 1 μM PP for 48 h (PP-treated BLS4 and control BLS4, respectively) and washed with PBS 3 times. Then, DLB1 cells (3 × 10^4^/ well) were cultured alone or co-cultured with control BLS4 or PP-treated BLS4 for 48 h. Viability was measured by an image analyzer after Hoechst and PI staining and plotted on a bar chart. (**B**) PP inhibited mitochondrial respiration in BLS4. Oxygen consumption rate (left panel) and extracellular acidification rate (right panel) were measured after the addition of 1 μM PP or 1 μM Rotenone, a complex I inhibitor and plotted on a bar chart as relative values to the values before treatment. The reduced oxygen consumption rate and the increased extracellular acidification rate indicated the inhibition of mitochondrial respiration and the compensatory facilitation of glycolysis, respectively. (**C**) PP induced ROS production in BLS4. After BLS4 was treated with or without 1 μM PP for 48 h, intracellular ROS was visualized by a fluorogenic probe, quantified as the fluorescent intensity and plotted on a bar chart. (**D**) PP treatment made the cellular redox balance in BLS4 oxidative. BLS4 was treated with PP as in (**C**). GSH/GSSG ratio was measured as in Materials and Methods and potted on a bar chart.

**Figure 5:**
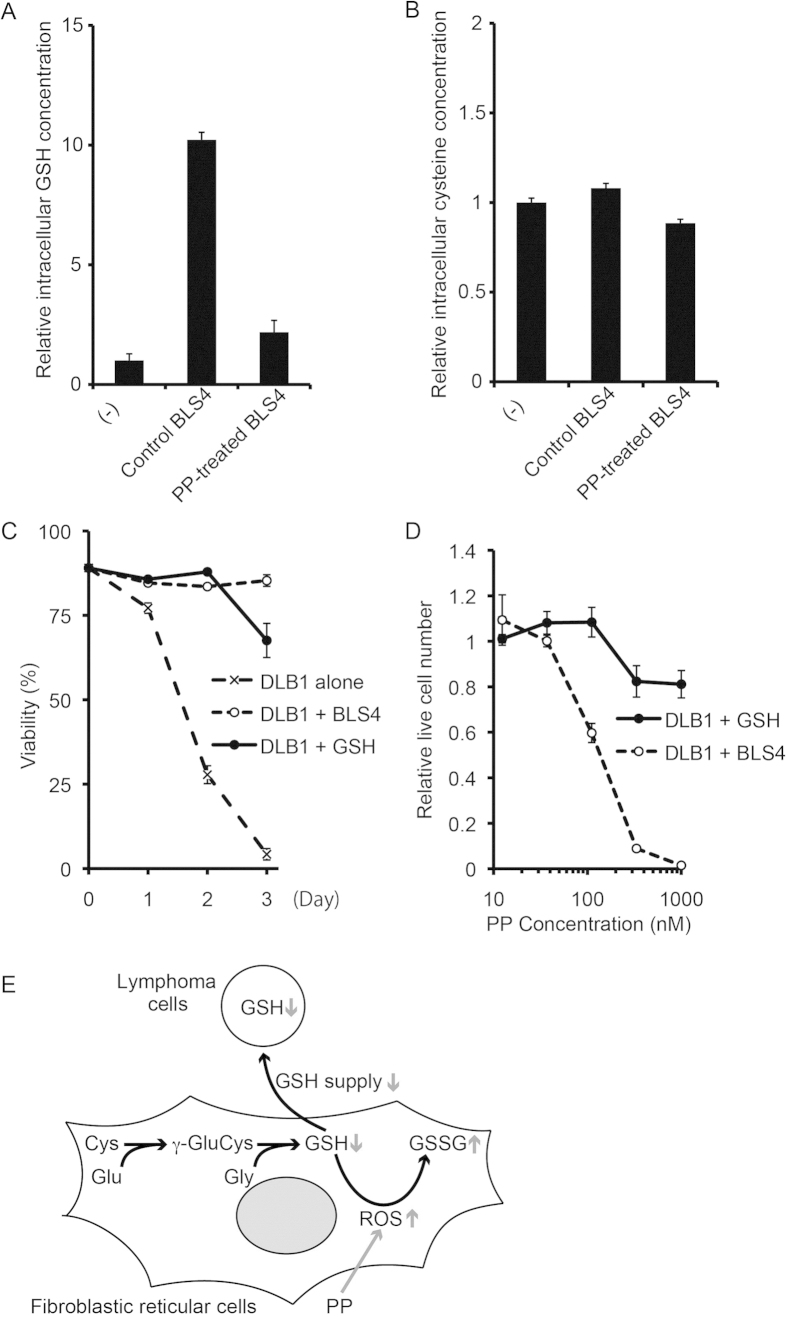
The mechanism of action of PP was the inhibition of GSH supply from BLS4 to PDX cells. (**A**) The intracellular GSH concentration of DLB1 cells was significantly increased by co-culture with control BLS4 but not by co-culture with PP-treated BLS4. DLB1 cells were cultured alone or co-cultured with control BLS4 or PP-treated BLS4 as in Fig. 4A for 24 h. Then, intracellular GSH concentration of DLB1 cells was measured by high-performance liquid chromatography as in Materials and methods and plotted on a bar chart. (**B**) The intracellular cysteine concentration of DLB1 cells was not affected by co-culture with BLS4. Intracellular cysteine concentration was measured as in (**A**). (**C**) GSH addition enabled *ex vivo* survival of DLB1 cells. DLB1 cells were monocultured with or without the addition of 2 mM GSH to the medium, or co-cultured with BLS4. Viability at the indicated time was measured as in Fig. 4A and plotted on a line graph. (**D**) GSH addition diminished PP-induced cell death. DLB1 cells monocultured in 2 mM GSH-containing medium or co-cultured with BLS4 were treated with the indicated dose of PP for 48 h. Live lymphoma cell number was counted as in Fig. 3C. (**E**) Schematic presentation of the putative model of the survival support by FRC and the mechanism of action of PP. PP-induced effects are indicated by a gray arrow.

We further investigated the mechanism of action of PP to reveal the tumor survival mechanism targeted by PDX cell-screening. We first tried to clarify whether the anti-tumor effect of PP was directly on lymphoma cells or mediated by an effect on BLS4. Surprisingly, BLS4 treated with PP for 48 hours lost the ability to support lymphoma cell survival even under PP-free conditions (Fig. 4A), although PP treatment did not affect BLS4 proliferative activity (Fig. 3C), suggesting that the anti-tumor effect of PP was mediated by a non-cytotoxic effect on BLS4. In order to investigate the speculation that BLS4 supported lymphoma cell survival by secreting certain cytokines or interacting with RN, we compared lymphoma cell survival among co-culture with BLS4, monoculture in conditioned medium of BLS4 and separated co-culture with BLS4 by Transwell. Unexpectedly, the separated co-culture could support lymphoma cell survival but the monoculture in conditioned medium could not, suggesting that lymphoma cell survival was supported by a relatively unstable soluble factor from BLS4, probably not cytokines (Supplemental Figure 3A). The reported mechanisms of action of PP are the inhibition of mitochondrial respiration, STAT3 signaling and Wnt signaling^17^,^18^. Because STAT3 and Wnt signaling were not detectably activated in BLS4 (data not shown), we investigated the effect of PP on mitochondrial respiration and found its inhibition by PP (Fig. 4B). We also compared the mRNA expression profile between PP-treated and non-treated BLS4 and the pathway analysis indicated the upregulation of glutathione (GSH) metabolism-related genes (Supplemental Figure 3B). GSH is a major cellular antioxidant, maintains cellular redox balance and protects cells from reactive oxygen species (ROS) stress. Therefore, we examined the effect of PP on ROS production and the cellular redox balance in BLS4. Strikingly, PP treatment strongly induced ROS production and reduced the ratio of GSH to oxidized glutathione (GSSG) and made the cellular redox balance oxidative in BLS4 (Fig. 4C,D). These results indicated that the inhibition of mitochondrial respiration by PP induced ROS production and altered the cellular redox status in BLS4. It has been recently revealed that bone marrow stromal cells modulate the redox status of chronic lymphocytic leukemia cells and promote cellular survival by providing cysteine, a precursor of GSH, for GSH synthesis^19^. Therefore, we speculated that the substance mediating survival support from BLS4 was GSH or its precursor and that PP inhibited its supply by increasing GSH consumption in BLS4. We then investigated cellular GSH concentration in lymphoma cells. Strikingly, it was markedly increased by co-culture with control BLS4, but not by co-culture with PP-treated BLS4 even under PP-free conditions (Fig. 5A). Of note, the cellular concentration of cysteine in lymphoma cells was hardly affected by co-culture with BLS4, indicating that BLS4 provided GSH, not cysteine (Fig. 5B). Consistently, supplying GSH to the culture medium could completely replenish the survival support of lymphoma cells by BLS4 (Fig. 5C). Furthermore, under GSH supply, lymphoma cell survival was hardly affected by PP treatment, indicating that the mechanism of action of PP was inhibition of GSH supply from BLS4 (Fig. 5D). The putative model of the survival support by FRC and the mechanism of action of PP are schematically demonstrated in Fig. 5E. The mechanism of action of PP was novel and unique, which suggested that PDX cell-screening could target different characteristics of cancer cells from conventional screening.

## Discussion

In this manuscript, we demonstrated a novel and important mechanism for lymphoma cell survival, the GSH supply from stromal cells. Cysteine supply for GSH synthesis from bone marrow stromal cells has been recently reported in chronic lymphocytic leukemia^19^. The fact that PP showed anti-tumor effect stronger than all 36 conventional anti-tumor drugs examined (Fig. 2C) indicated the high potential of the therapeutic strategy targeting the GSH supply and the usefulness of the PDX screening for the drug development.

Cell lines have been used for many years as drug discovery tools. They have contributed to drug development as simple and useful models of cancers and produced great results; however, the limitations of cell line-based screening have come to light. Cell lines do not recapitulate real cancers faithfully in some aspects, especially in the points of microenvironment independency and rapid growth rate^6^,^8^. Only rare cases of cancer have genetic mutations that confer a particularly strong survival advantage on cancer cells and can survive microenvironment independently to be a cell line. Probably due to the natural selection of rapid-growing clones during *ex vivo* culture and the secondary effect of genetic mutations that enable microenvironment-independent survival, cell lines have a tendency to grow much faster than primary cancers. Therefore, using cell lines for drug screening leads to an overestimation of the rapid cell cycle progression of cancer cells as a therapeutic target and a disregard of microenvironmental support for cancer survival. Consequently, the majority of conventional anti-cancer drugs have mechanisms of action targeting rapid-growing cells such as inhibition of the copy, synthesis or repair of DNA and inhibition of the polymerization or depolymerization of microtubules; however, primary cancer cells, especially cancer stem cells, grow more slowly than cell lines and will be less sensitive to these drugs. In addition, almost no conventional drug targets microenvironmental support, although most cancer cells depend on it. Without using a new model recapitulating the phenotypes of primary tumor instead of cell lines, it seems to be difficult to develop innovative anti-cancer drugs by cell-based screening systems from now on.

PDX are established by the transfer of primary cancer cells directly from patients into immunodeficient mice. PDX are maintained by passage of tumor cells directly from mouse to mouse. They maintain the characteristics of the parental tumors faithfully^6^. Detailed examination indicates that when passaged in mice, PDX are biologically stable in terms of histology^20^, gene expression profiles^21^, mutational status including genome copy number variants^20^,^22^,^23^, metastatic potential^20^, drug responsiveness^24^,^25^ and hierarchy of the cancer cell differentiation status^26^,^27^. In fact, our lymphoma xenografts retained high similarity of gene expression profiles (Supplemental Table 2). In the previous report, the correlation coefficient of gene expression profiles between 3 PDX cell-derived cell lines and their original PDX cells was 0.674^28^. The correlation coefficients between original patients and PDX cells were 0.814 to 0.890 (Supplemental Table 2), suggesting that PDX cells maintained much greater similarity of the gene expression profile than cell lines. Furthermore, co-culture with BLS4 maintained the gene expression profiles of DLB1 cells almost completely, with a correlation coefficient of 0.958, for the required period for the screening (Supplemental Figure 2). Therefore, PDX cell-screening succeeded in applying cells highly similar to primary cancer cells to high throughput drug screening.

PDX has been used for analyses of cancer-initiating cells^26^,^27^, investigation of biomarkers^29^, evaluation of the efficacy of preclinical drugs^25^ and prediction of the sensitivity of original patients to conventional anti-tumor drugs for patient-specific therapy^24^,^30^; however, they have never been used for a high throughput drug-screening system using thousands of compounds. By introducing the element of phenotype of primary cancer cells at earlier *in vitro* stages of drug development, PDX cell-screening allows us to find drugs that are active against real cancers *in vivo* and bridges the gap between the oversimplicity of conventional cell line-based screening and the intractable complexity of clinical studies.

There are some limitations for using PDX in an anti-cancer drug screening. For examples, not all primary cells could engraft in mice, especially cells of some diseases such as multiple myeloma and chronic myeloid leukemia in chronic phase are extremely hard to engraft. In addition not all PDX cells can survive *ex vivo* even when they are co-cultured with BLS4 or other stromal cells. For reliable estimation of anti-tumor effect of compounds, the viabilities of the untreated (control) PDX cells need to be more than 50% on Day6 of the screening. Two out of four PDX cells could satisfy this condition in our study (Fig. 1A). In addition, DLB2 cells had a tendency to make aggregation, which made the cell count by the image analyzer difficult. As a result, we could establish only one PDX cell-screening out of 4 PDX. PDX cell-screening is available for only limited disease and patients at present.

It should be noted that many of the 36 conventional anti-tumor drugs included in the library did not demonstrate high cytotoxicity to DLB1 cells (Fig. 2C and Supplemental Table 4), which was consistent with our individual investigation using 5-fluorouracil and etoposide (Fig. 1C). One reason is DEI considering the cytotoxic effect on BLS4; however, even in the comparison by only cytotoxic effect on PDX cells, the cytotoxicity of the conventional anti-tumor drugs was not very high. The top 4 drugs were bortezomib, bleomycin, oxaliplatin and paclitaxel that ranked 33rd, 65th, 72nd and 73rd, respectively. And more than half (19) of the drugs could not rank in the top 25% (data not shown). This could be because DLB1 cells obtained from the relapsed patient at the terminal stage, or because the concentration of all the compounds was 2 μM in this screening, which might not be enough for some compounds; however, DLB1 cells demonstrated higher resistance to anti-tumor drugs at multiple concentrations than U-2932 that established from the patient after multiple relapses (Fig. 1C)^31^. Therefore, it is possible that conventional anti-tumor drugs selected by cell line-based screenings were not as cytotoxic for primary tumor cells as they were for cell lines. And this might be the reason many lymphoma patients are refractory to chemotherapies or relapse after chemotherapies. Conversely, there are many compounds demonstrating higher cytotoxicity than conventional anti-tumor drugs in our screening, it is possible that PDX screening discover innovative anti-tumor drugs overlooked by cell line-based screenings.

In this study, we could establish only one PDX cells that was suitable for a high throughput screening; therefore, PP was selected by only one PDX cell-screening although it demonstrated strong anti-tumor effect on another lymphoma PDX. Taking the low bioavailability and the toxicity of PP into consideration, PP itself will not be a promising anti-lymphoma drug for human; however, the important things are the facts that PDX cells could be used for high throughput screening and that PDX cell-screening could pick up a drug with a unique mechanism of action. We established 3 lymphoma PDXs from 4 DLBCL patients and one PDX cells from them was suitable for high throughput screening. It will not be difficult to establish more DLBCL PDX cells suitable for high throughput screening and our system is easy to scale up to screen more than a hundred thousand compounds. It seems possible to find compounds that demonstrate strong cytotoxicity to multiple DLBCL PDXs and have good bioavailability with a large scale PDX cell-screening although it requires a large scale compound library and a lot of cost and labor. They will be promising anti-tumor drugs for DLBCL.

In summary, we developed a PDX cell-screening system applying primary-like tumor cells obtained from PDX to high throughput *in vitro* screening, discovered the GSH supply as an important mechanism of stromal support for lymphoma survival and identified PP as its inhibitor. By introducing the phenotype of primary cancer cells at earlier *in vitro* stages of drug development, PDX cell-screening sheds new light on anti-cancer drug development.

## Methods

### Cells and reagents

SU-DHL4 and U-2932, cell lines of DLBCL, were purchased from American Type Culture Collection (Manassas, VA) and Deutsche Sammlung von Mikroorganisem und Zellkulturen (DSMZ) (Braunschweig, Germany), respectively. Both cell lines were cultured in Roswell Park Memorial Institute medium (RPMI) 1640 supplemented with 10% fetal bovine serum (FBS). BLS4 was described previously^11^,^13^ and was cultured in 10% FBS-containing Dulbecco’s modified Eagle’s medium (DMEM) for maintenance and in 10% FBS-containing RPMI 1640 for all experiments. DLB1 cells were cultured with BLS4 in 10% FBS-containing RPMI 1640. They were stripped with pipetting and re-seeded on newly prepared BLS4 once a week. For DLB1 monoculture in conditioned medium of BLS4, conditioned medium was prepared as follows: BLS4 (5 × 10^4^/well of a 6-well plate) was cultured as above for 4 days and the medium was collected and centrifuged to remove floating cells. For separate co-culture of DLB1 cells with BLS4, a Transwell (Corning Inc., Corning, NY) was used.

PP was purchased from Sato Pharmaceuticals Co. Ltd (Tokyo, Japan). GSH and Rotenone were from Wako Chemicals (Osaka, Japan). Calsein-AM and propidium iodide (PI), DAPI and Hoechst and Annexin V were obtained from Dojindo Laboratories (Kumamoto, Japan), Life Technologies (Carlsbad, CA) and Roche Applied Science (Penzberg, Germany), respectively.

Prestwick and Lopack chemical library was provided by the Open Innovation Center for Drug Discovery (The Tokyo University, Tokyo, Japan). These libraries are also commercially available at Prestwick Chemical (Strasbourg, France) and Sigma-Aldrich (St. Louis, MO), respectively. Detailed information about the library are available at their web sites, http://www.prestwickchemical.com/index.php?pa=26 and http://www.sigmaaldrich.com/catalog/product/sigma/lo1280?lang=en&region=US, respectively.

### Antibodies

Anti-human CD45 antibody and anti-mouse CD45 antibody were from BD Biosciences (San Jose, CA).

### Establishment of lymphoma PDX

Primary lymphoma cells from patients were collected after obtaining written informed consent, preserved and transplanted into NOG mice as described previously^27^, except that cells were injected intraperitoneally. Lymphoma cells were collected from patients with informed consent. Patients’ characteristics are shown in Supplemental Table 1. This study was approved by the institutional review board of Nagoya University Graduate School of Medicine and performed in accordance with the ethical guideline for clinical studies issued by Japanese government. The use of mice in this study was permitted by the Animal Care and Use Committee of Nagoya University Graduate School of Medicine and carried out in accordance with its guideline.

### Subcutaneous inoculation of tumor cells

DLB1 or DLB2 cells (5 × 10^6^) together with BLS4 (2 × 10^5^) were subcutaneously inoculated into NOG mice. Tumor volume was calculated using the following formula: Tumor volume (mm^3^) = (d^2^ × D)/2, where D (mm) and d (mm) are the longest and shortest diameters of the tumor, respectively.

### Microarray

Microarray was performed by Filgen Inc. (Nagoya, Japan), according to their standards.

### Cell proliferation assay

Cell proliferation was analyzed by the MTT assay using Cell Count Reagent SF (Nacalai Tesque, Kyoto, Japan) according to the manufacturer’s instructions.

### Immunohistochemistry, immunofluorescence and flow cytometry

These were performed as described previously^27^,^32^.

### PDX cell-screening

Day 1: BLS4 (3 × 10^3^ cells/well) was seeded in 96-well plates. Day 2: DLB1 cells were obtained from sacrificed DLB1 mice and seeded (3 × 10^4^ cells/well) in the BLS4-seeded plates. Day 3: Library compounds (2 μM each) were added to the cell culture. Day 6: Dead cells were stained with DAPI. Dead lymphoma cells were selectively counted with an ArrayScan VTI HCS Reader (Thermo Scientific, Yokohama, Japan). For evaluation of the effect on BLS4 proliferation, BLS4 was seeded in the same way on day 1, treated with the same library compounds on day 3 without seeding DLB1 cells and subjected to MTT assay on Day 6.

### Measurement of mitochondrial respiration

Oxygen consumption rate and extracellular acidification rate were measured by Extracellular Flux Analyzer XFe96 (Seahorse Bioscience, Billerica, MA) according to the manufacturer’s instruction.

### Measurement of ROS production in cells

ROS production was visualized by a fluorogenic probe (CellRox Deep Red Reagent, Life Technologies) and observed by a fluorescence microscope, In Cell Analyzer 6000 (GE, Fairfield, CT). The fluorescent intensity of the images was quantified by the attached software.

### Measurement of cellular GSH/GSSG ratio, GSH concentration and cysteine concentration

The GSH/GSSG ratio was calculated using the GSH/GSSG-Glo Assay kit (Promega, Madison, WI), according to the manufacturer’s instruction.

Intracellular GSH and cysteine concentration were measured as described previously^33^. Briefly, cells were lysed by ice-cold lysis buffer (NaCl 68.5 mM, KCl 1.35 mM, Na_2_HPO_4_ 5 mM, KH_2_PO_4_ 0.88 mM, pH 7.4) and GSH and cysteine were derivatized with ABD-F (Dojindo Laboratories, Kumamoto, Japan). The derivatized GSH and cysteine were subjected to high-performance liquid chromatograpy with a YMC-Triart C18 column (YMC CO., LTD., kyoto, Japan). Separation was achieved by a mobile phase of Buffer A (20 mmol/L phosphate buffer (pH6.5)) and buffer B (acetonitrile/methanol/H_2_O [45:40:15, v/v/v] at a flow rate of 0.4 mL/min. ABD-F-derivatized GSH and cysteine were detected by a fluorescence detector (ex380/em510) and quantified from the peak area using a standard curve.

## Additional Information

**How to cite this article**: Sugimoto, K. *et al*. Discovery of a drug targeting microenvironmental support for lymphoma cells by screening using patient-derived xenograft cells. *Sci. Rep*. **5**, 13054; doi: 10.1038/srep13054 (2015).

## Supplementary Material

Supplementary Information
