# Supplementary material for: Discovery of a drug targeting microenvironmental support for lymphoma cells by screening using patient-derived xenograft cells
Source: Sci Rep. 2015 Aug 17;5:13054. doi: 10.1038/srep13054 (PMC4538400; doi:10.1038/srep13054)
Supplement: Supplementary Information [file srep13054-s1.doc]

**Discovery of a drug targeting microenvironmental support for lymphoma cells by screening using patient-derived xenograft cells**

Keiki Sugimoto^1,2^, Fumihiko Hayakawa^1^*, Satoko Shimada^3^,

Takanobu Morishita^1^, Kazuyuki Shimada^1^, Tomoya Katakai^4^, Akihiro Tomita^1^, Hitoshi Kiyoi^1^, Tomoki Naoe^1,5^

^1^ Department of Hematology and Oncology, Nagoya University Graduate School of Medicine, Nagoya, Japan

^2^ Fujii Memorial Research Institute, Otsuka Pharmaceutical Co., Ltd., Otsu, Japan

^3^ Department of Pathology and Clinical Laboratories, Nagoya University Hospital, Nagoya, Japan

^4^ Department of Immunology, Niigata University Graduate Scholl of Medical and Dental Sciences

^5^ National Hospital Organization Nagoya Medical Center, Nagoya, Japan

*Corresponding author

Department of Hematology and Oncology

Nagoya University, Graduate School of Medicine,

65 Tsurumai-cho, Showa-ku, Nagoya, 466-8550, Japan

Tel.: 81-52-744-2145

Fax: 81-52-744-2161

e-mail: [bun-hy@med.nagoya-u.ac.jp](mailto:bun-hy@med.nagoya-u.ac.jp)


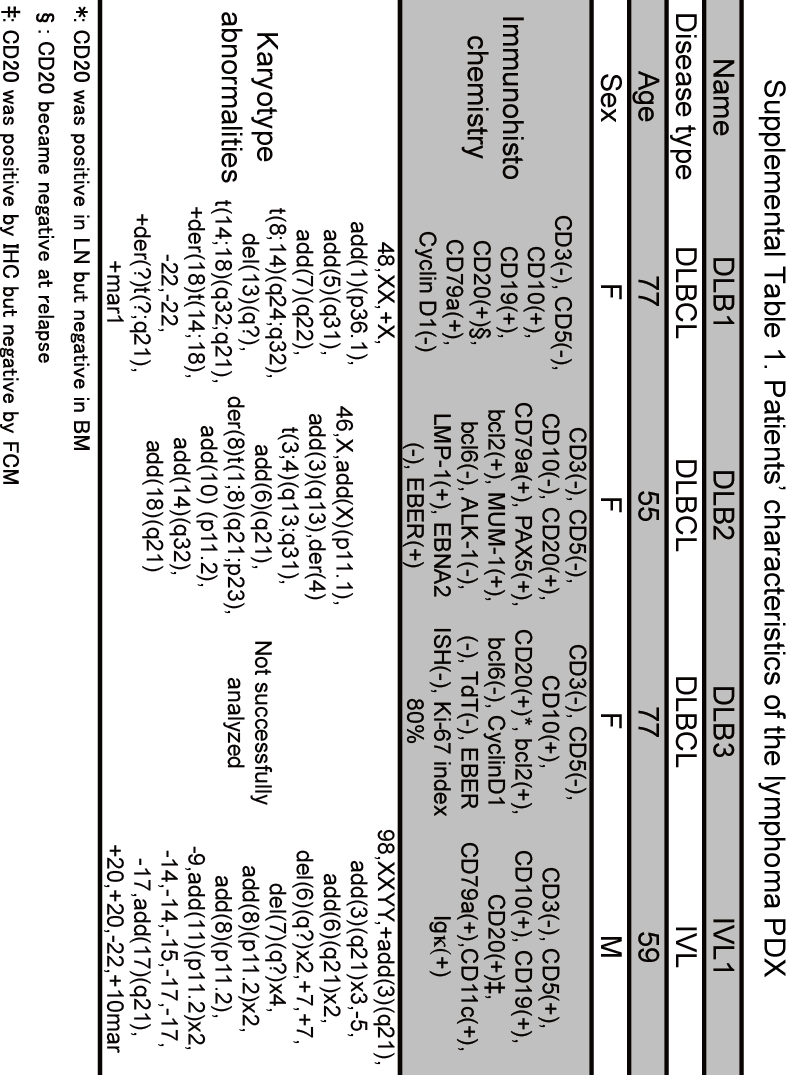


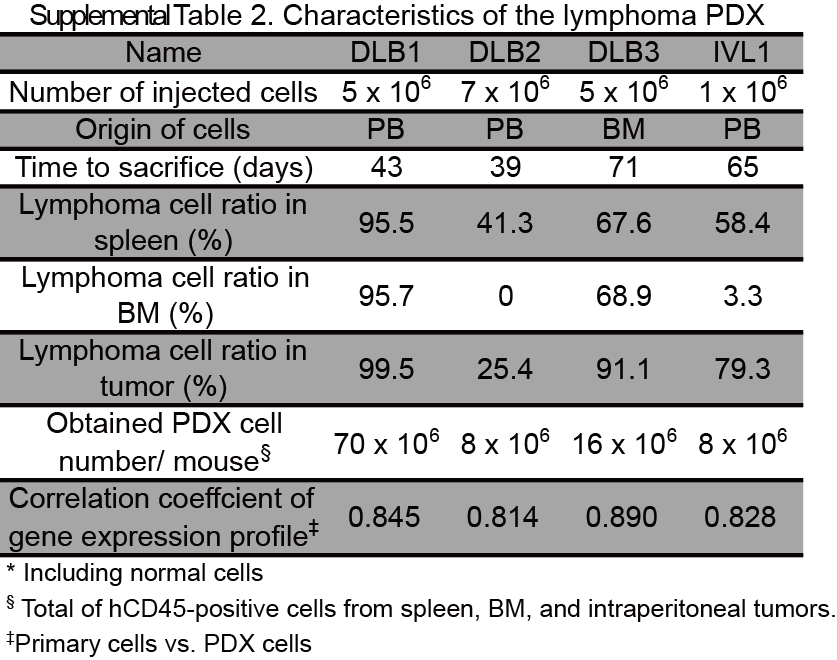


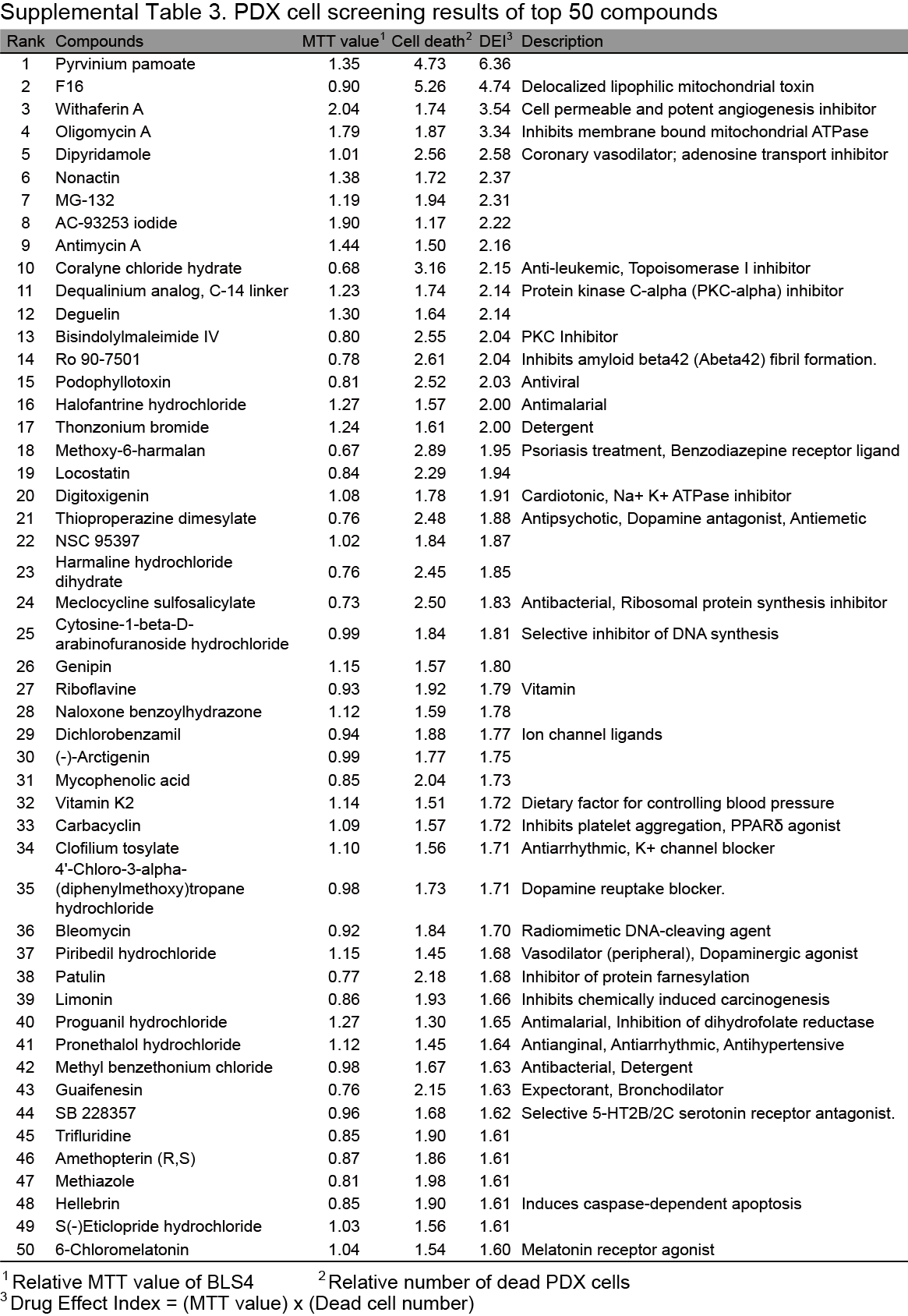


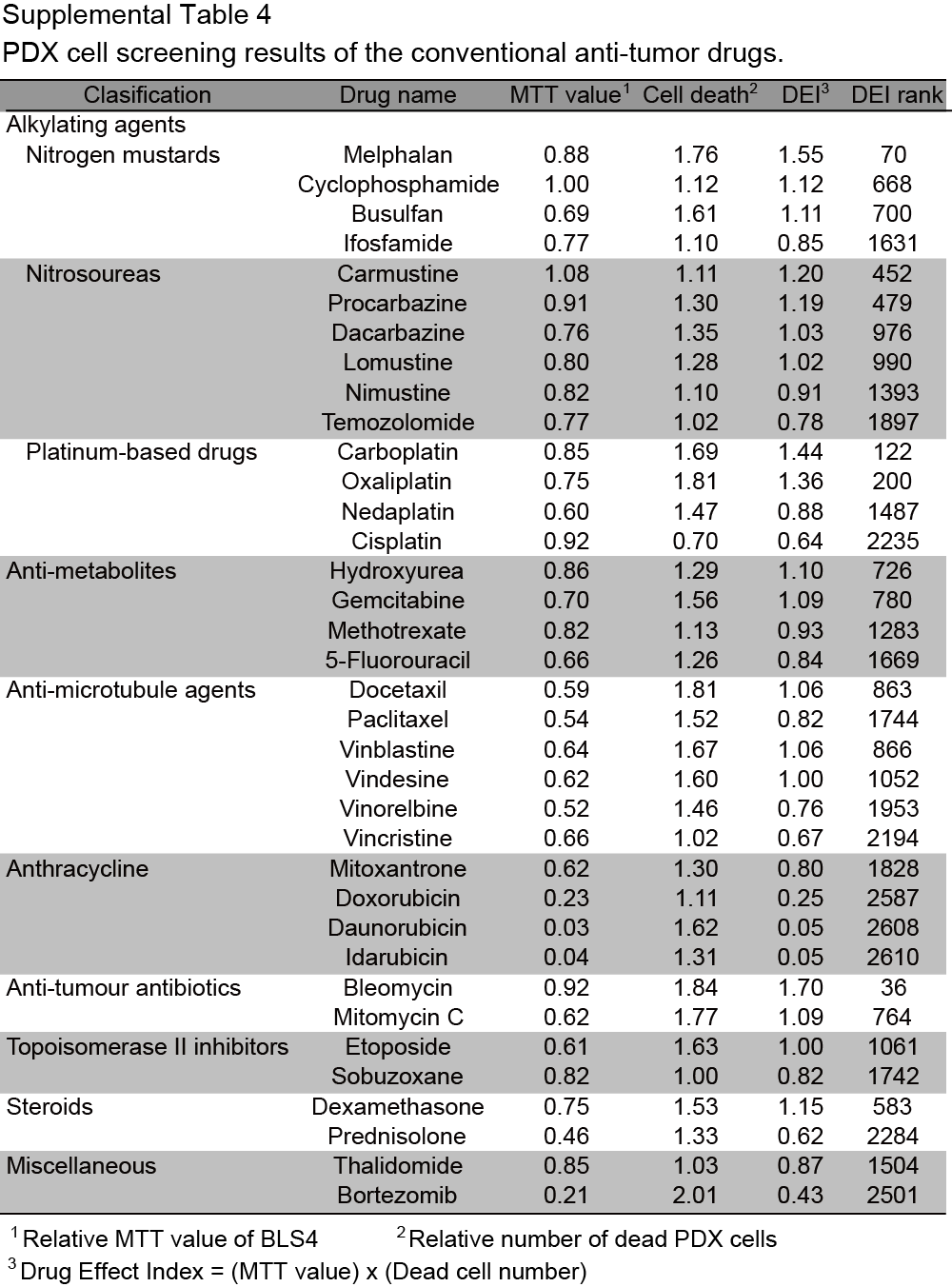


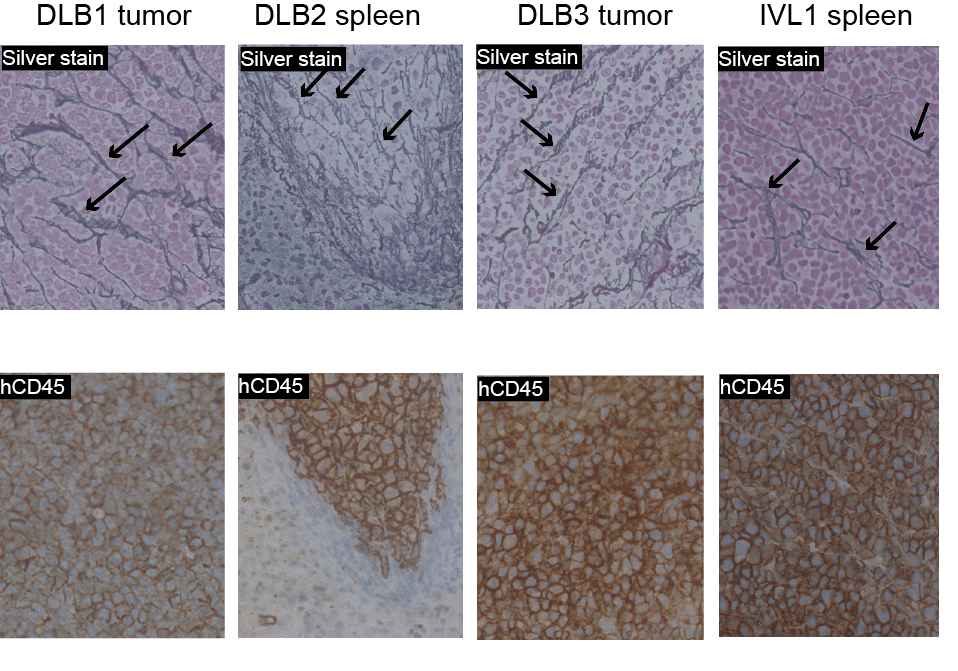


**Supplemental Figure 1** RN formation in tumor of lymphoma PDX. Sections of the spleen or intraperitoneal tumor of the indicated PDX were stained by silver stain (upper panel) and immunostained by anti-human CD45 antibody (lower panel). Reticular fibers are indicated by arrows.


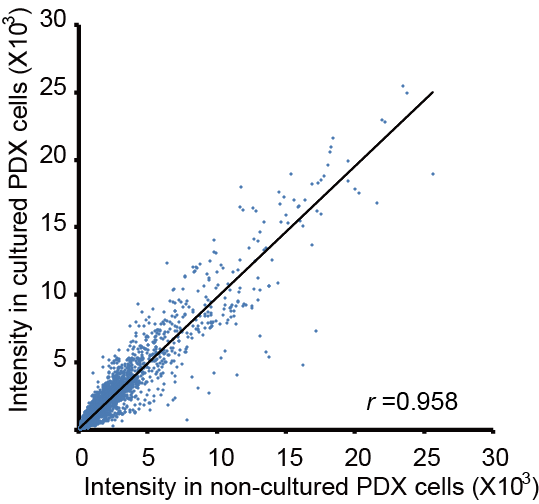
**Supplemental Figure 2** Co-culture with BLS4 maintained the global gene expression profile of DLB1 cells almost completely. Microarray was performed using mRNA of PDX cells just after collection from PDX (non-cultured PDX cells) and that of PDX cells after ex vivo culture with BLS4 for 4 days (cultured PDX cells). The 27327 gene with a good quality flag was plotted on a scattergram where signal intensities in cultured PDX cells and non-cultured PDX cells were set on the Y-axis and X-axis, respectively. The linear trendline and correlation coefficient (*r*) are shown.


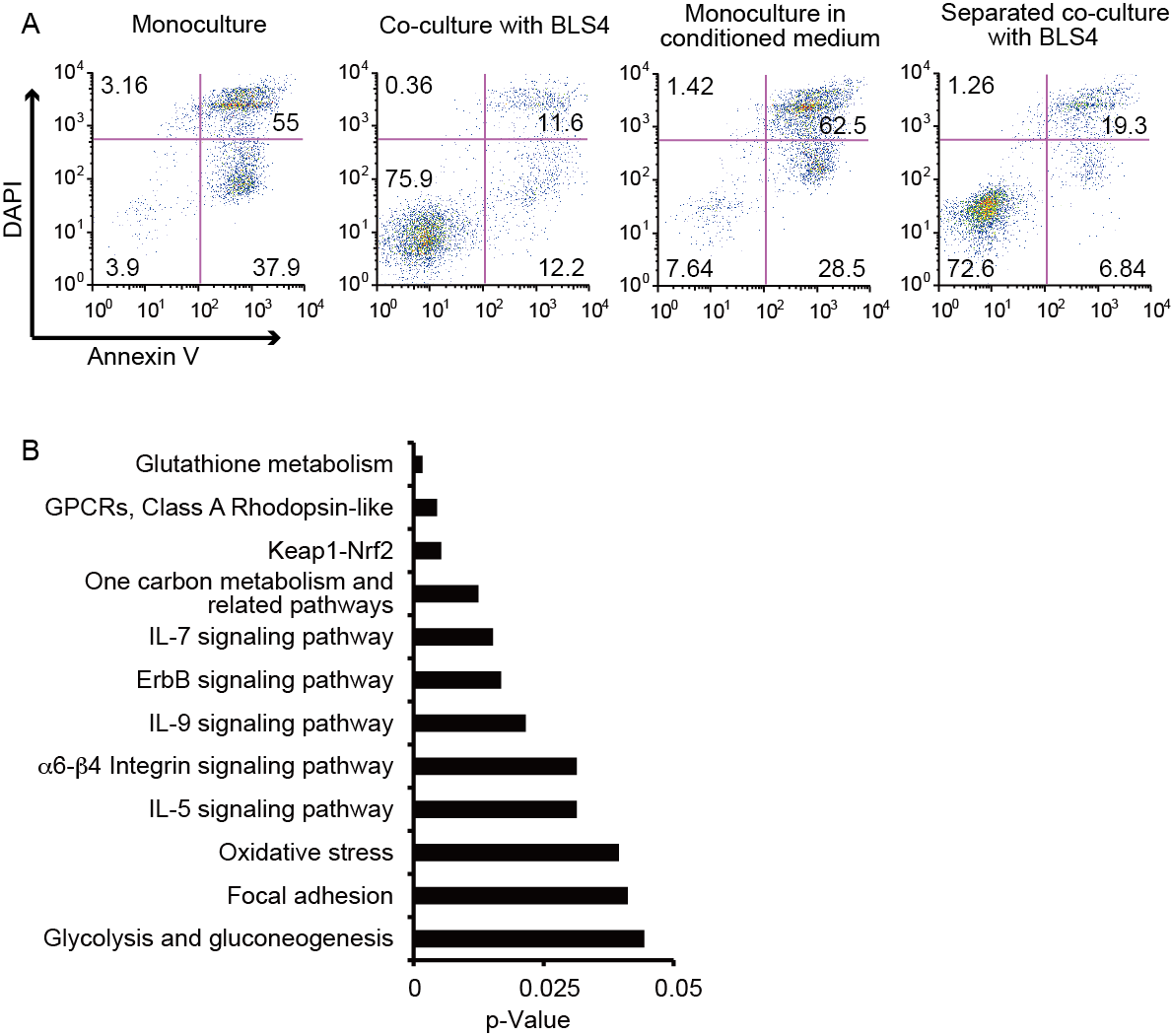


**Supplemental Figure 3** (A) Search for the factor required for BLS4 to support PDX cell survival. DLB1 cells were monocultured, co-cultured with BLS4, monocultured in conditioned medium of BLS4, and co-cultured with BLS4 with separation by Transwell for 48 h. Cells were stained with DAPI and annexin V and analyzed by flow cytometer. The separated co-culture could support lymphoma cell survival but the monoculture in conditioned medium could not. (B) Pathways that may be related to PP treatment reaction. Microarray was performed using mRNA extracted from BLS4 with or without treatment with 1 µM PP for 48 h. Gene expression profiles were compared between them and functional pathway analysis was performed using DAVID software and the Kyoto Encyclopedia of Genes and Genomes (KEGG). A bar chart of the p-value of the top 12 pathways with low p-value is demonstrated. The pathway having the highest possibility of being related with the PP treatment reaction was the glutathione metabolism pathway.
